# Complete Genome Sequence of Lactococcus lactis subsp. lactis G50 with Immunostimulating Activity, Isolated from Napier Grass

**DOI:** 10.1128/genomeA.00069-18

**Published:** 2018-02-22

**Authors:** Kazuma Nakano, Maiko Minami, Misuzu Shinzato, Makiko Shimoji, Noriko Ashimine, Akino Shiroma, Shun Ohki, Tetsuhiro Nakanishi, Hinako Tamotsu, Kuniko Teruya, Kazuhito Satou, Naoko Moriya, Hiromi Kimoto-Nira, Miho Kobayashi, Tatsuro Hagi, Masaru Nomura, Chise Suzuki, Takashi Hirano

**Affiliations:** a Okinawa Institute of Advanced Sciences, Uruma, Okinawa, Japan; b Institute of Livestock and Grassland Science, NARO, Tsukuba, Ibaraki, Japan

## Abstract

Lactococcus lactis subsp. lactis G50 is a strain with immunostimulating activity, isolated from Napier grass (Pennisetum purpureum). We determined the complete genome sequence of this strain using the PacBio RS II platform. The single circular chromosome consists of 2,346,663 bp, with 35.03% G+C content and no plasmids.

## GENOME ANNOUNCEMENT

Strains of Lactococcus lactis are widely used as starters for manufacturing fermented dairy products and fermented vegetables and are also found in various natural environments (1, 2). Because L. lactis is not a natural inhabitant of the mammalian gastrointestinal tract, it had not generally been regarded as a probiotic. Several natural isolates with beneficial health properties, however, have been described (3–5), and some strains are able to survive in the gastrointestinal tract (6–8). We previously investigated the potential use of L. lactis strains as probiotics (7, 9).

L. lactis subsp. *lactis* G50 was isolated from Napier grass (Pennisetum purpureum Schumach). Among 15 L. lactis strains, G50 induces the highest production of cytokines (interleukin 12 [IL-12], IL-6, and tumor necrosis factor α [TNF-α]) in the macrophage-like cell line J774.1 (10). Heat-treated G50 loses the ability to induce TNF-α in J774.1 (11). *In vivo*, oral administration of live G50 cells reduces the total IgE antibody production in ovomucoid-sensitized BALB/c mice (10). Long-term oral administration of heat-treated G50 cells to senescence-accelerated mouse prone 6 (SAMP6) does not suppress senescence-associated changes. However, fecal IgA levels of G50-fed mice (3-month-old SAMP6 mice given G50 cells for 2 months) were higher than those of control mice, and the intestinal growth of H_2_S-producing bacteria was suppressed in G50-fed mice (12).

Genomic information is key to clarifying the potential functions of the strain. To identify potential genetic determinants specifying the properties of strain G50, we determined the complete genome sequence using single-molecule real-time (SMRT) technology (13). SMRT technology offers advantages such as long read lengths, high consensus accuracy, and a low degree of bias and is a powerful tool for sequencing complete bacterial genomes with highly repetitive sequences (14, 15).

The genomic DNA was purified at the early log phase using a PowerClean DNA cleanup kit (Mo Bio Laboratories, Carlsbad, CA), followed by 20-kb library construction for P6-C4 chemistry with shearing (15). Two SMRT cells (each a 240-min movie) were used for sequencing on the PacBio RS II platform (Pacific Biosciences, Menlo Park, CA). *De novo* assembly was performed using the Hierarchical Genome Assembly Process 2 workflow (16). A single circular contig representing one chromosome (2,346,663 bp, G+C content of 35.03%, and 925× coverage) was obtained. No plasmid was detected. PacBio RS II sequencing produced 654,840 reads, with an average length of 4,305 bp, a maximum length of 49,160 bp, and uniform coverage.

The complete genome sequence of Lactococcus lactis subsp. *lactis* G50 will help to elucidate the immunostimulating mechanism and will provide insight into the diversity among Lactococcus lactis strains.

### Accession number(s).

The complete genome sequence of Lactococcus lactis subsp. lactis G50 has been deposited in DDBJ/ENA/GenBank under the accession number CP025500.
